# Studying the Effects of Mobile Phone Waves on Electro Cardiogram Parameters of Students in Zahedan University of Medical Sciences

**DOI:** 10.5812/ijhrba.4562

**Published:** 2012-07-25

**Authors:** Gholamreza Komeili, Sima Nabizadeh Sarabandi

**Affiliations:** 1 Department of Physiology, School of Medicine, University of Medical Sciences, Zahedan, IR Iran; 2 Department of English, School of Medicine, University of Medical Sciences, Zahedan, IR Iran

**Keywords:** Mobile Phone, Electrocardiogram, Students, Iran

## Abstract

**Background:**

The increasing use of mobile phones in recent years has caused concerns about the effects of electromagnetic waves of mobile phonesonhuman biological processes.

**Objectives:**

This study was conducted in order to survey the effects of mobile electromagnetic waves on electro cardiogram parameters as heart rate, TP segment, PR interval, Time of QRS and T waves, and voltage of R wave.

**Patients and Methods:**

In this quasi experimental study, 40 students, of Zahedan medical science University, 20 boys and 20 girls, who had referred to the laboratory of physiology were selected. At first a normal electro cardiogram in lead I was recorded for each of the subjects for 20 seconds. Then a mobile phone was placed near their body and while mobile was ringing and talking two other electrocardiograms were recording for 20 seconds. Electro cardiograms were recorded with power lab device and analyzed by chart 5 software. Finally an ANOVA was employed to analyze the data through the SPSS 17, followed by a Tukey test.

**Results:**

There was significant difference between heart rate during talking in comparison with heart rate during ringing and resting in both genders. There was a significant decrease of resting TP segment in comparison with TP segment during ringing and talking in males whereas in females TP segment indicated significant difference in all three conditions. There was a significant increase in T wave time in males during talking in comparison with resting and ringing; however there was no significant difference in that of females in any of the three stated conditions. This study revealed that there is not any significant difference in PR interval, Time of QRS wave and R wave voltage.

**Conclusions:**

According to the results of this study, mobile phones can affect the heart rate, TP segment and time of T wave. Therefore, it seems that long term use can affect heart. Based on several reports on the effects of these waves on biological processes, precautionary measures should be taken about using mobile phones.

## 1. Background

The spectrum of electromagnetic waves has an extensive frequency range from 300 MHz to 300 GHZ and their wave lengths vary from 1 mm to 1 m. Waves emitted by mobile phones with an average frequency of 900 MHz to 1 GHz are also in this frequency range (1). The developing and increasing use of mobile phones which produce electromagnetic waves and several reports during the recent years on tratogenic effects of these waves on different growth processes have caused concerns about human health. Extensive increase of microwave producing devices such as mobile phones have drawn biological researchers’ attention to study their effects on human health (2). Some surveys have reported signs as sleeping, disturbance, headache, anxiety, depression and fatigue among people who live in the mobile aerials surroundings whereas in other studies no relationship between these signs and mobile wave radiations has been recorded. The results of some epidemiologic studies revealed that mobile waves with a power density lower than (1mv/cm^2^) can cause signs such as headache, heat sense in the ear, memory weakness and fatigue, there is also significant relationship between length and numbers of conversations during a day and onset of signs (3). Andrzejak and colleagues studied the effects of mobile phone on the Heart Rate variability (HRV) of the students who had natural electrocardiogram and echocardiography. Variations of HRV before, during and after, talking by mobile were recorded. The result indicated significant difference of HRV during ringing and after talking in comparison to before talking (4). Another study was conducted to survey the effects of mobile phone on heart rate and blood pressure in rat, but no significant difference in heart rate and blood pressure was observed (5). The studies about the effect of electromagnetic waves on the brain indicated that the identical global system waves of mobile phone increase the neural activities of the brain in 52% and decrease the same in 17% of subjects. Similar studies suggested that 700 MHz magnetic field can cause upsetting in electrical activities of hippocampus of mouse brain (6). In a case control study, long term use of mobile phone on brain tumor was studied. In this study, after ten years follow up, the results in the United State of America (USA) and 5 European countries indicated that constant mobile phone users are not in a higher risk of brain tumor compared to the people who never or rarely use mobile phones (7-9). Another research was conducted to study the effects of mobile phone electro wave on short time memory, concentration and spatial memory of mice and the results indicated a decrease in animals’ spatial memory (3). Considering the findings of previous studies on the harmful effects of mobile phone waves on one hand and the inevitable need to use mobile phones in daily life on the other hand, this research was conducted to study the effects of mobile phone waves on electrocardiogram in healthy young people. It should be stated that few studies have been conducted on the effects of these waves on heart.

## 2. Objectives 

This research was this study was aimed to find out the effects of mobile electromagnetic waves on electro cardiogram parameters such as heart rate, TP segment, PR interval, Time of QRS and T waves, and voltage of R wave.

## 3. Patients and Methods

This quasi experimental study was done on 40 students, 20 boys and 20 girls, of Zahedan University of medical sciences who referred to physiology laboratory in winter 2012. The samples were chosen by incidental sampling according to the study criteria. The study objectives were explained to the students and they contributed to the study sensibly and deliberately. The criteria included: 1/student of Zahedan university of medical science, 2/ regular use of mobile. The excluding criteria were: abnormal electrocardiogram, using heart medicine, having cardiovascular disease such as hypertension, congenital heart disease, heart operation background and metabolic and neurologic disturbances. At first a normal electrocardiogram in lead I for 20 seconds was recorded for each of the subjects. Then a mobile phone was placed near their body, while ringing and talking, two other electrocardiograms were recorded for each subject, each of them for 20 seconds. Electrocardiogram recording was done by power lab recording device, finally the collected data were analyzed by chart 5 software. In addition, pulse transducer was connected to the index finger of the subjects to record their heart rate during all procedures. It should be stated that the identical mobile phone (Nokia 5220) was used for all subjects. The number of heart rate, TP segment, time of T wave, PR interval, time of QRS of the subjects and the results were recorded in an information form for all of the individuals. Analyzing method: The recorded data in the information form were transferred into the computer in order to compare the differences before mobile phone ringing, during ringing and during talking. An ANOVA was employed to analyze the data by SPSS 17 software followed by the Tukey test. The values *P* < %5 were considered significant.

## 4. Results

**Figure 1.**
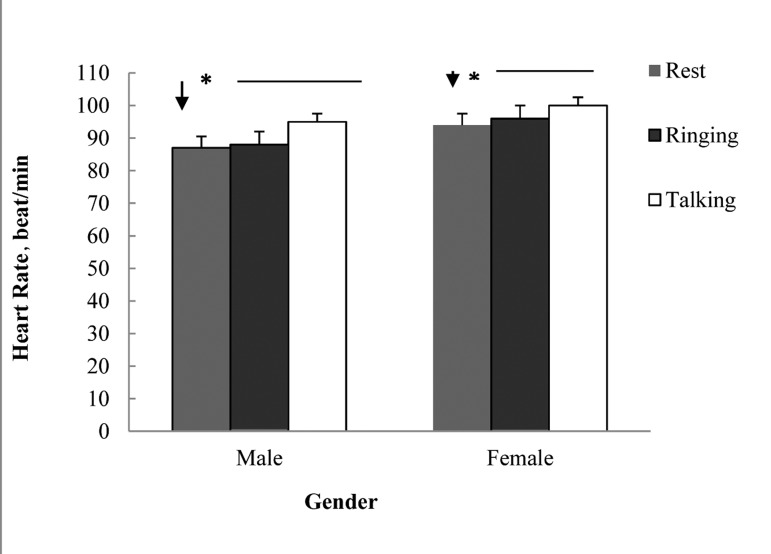
Effects of Mobile Phone Talking on Heart Rate in Studied Groups * *P* < 0.05

**Figure 2.**
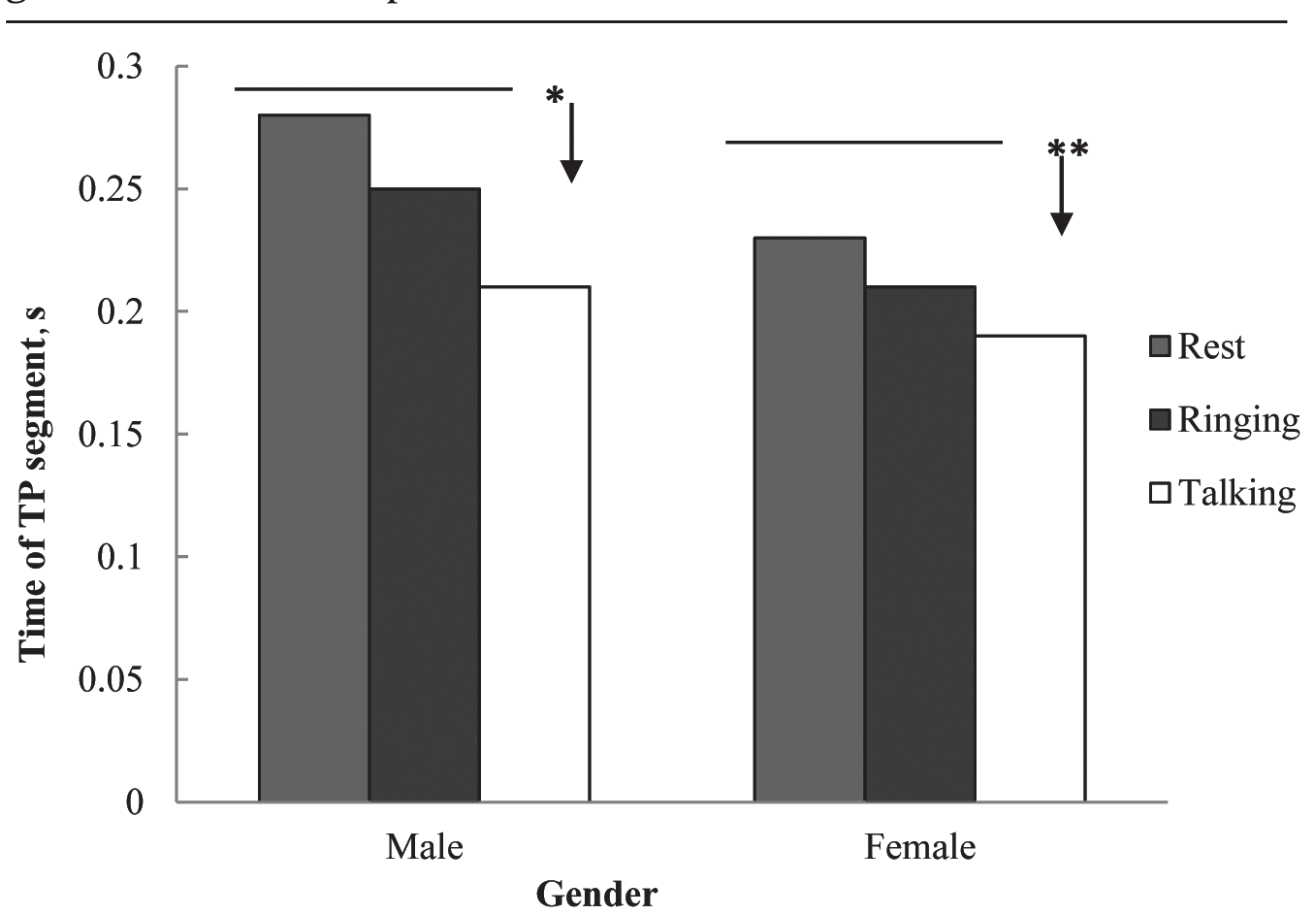
Effects of Mobile Phone Talking on TP Segment of Electrocardiogram in Studied Groups *P < 0.05, **P < 0.01

As indicated in *Figure 1*, talking by mobile caused significant increase in heart rate mean of the subjects under study (both girl and boy groups) in comparison with resting state (before ringing) and during ringing (*P* < 0.01). TP segment had a significant decrease during talking by mobile in comparison with resting and ringing states in both female (*P* < 0.01) and male (*P* < 0.05) (*Figure 2*). There wasn’t any significant difference in RP interval in the three states (resting, ringing and talking) in both groups. Significant increase in time of T wave during talking in comparison with resting and ringing in males (*P* < 0.01) was observed whereas in female there was no significant difference (*Table 1*). Talking through mobile phone didn’t have any significant effect on voltage of R wave in both female and male groups (*Table 2*), also there wasn’t any significant difference in time of QRS wave in any of the groups under study (*Table 3*).

**Table 1.** The Comparison of T Wave Time of Electrocardiogram of Different Studied Groups (*P* value for Comparing Resting State With Talking Time)

	Mean, s	Standard Deviation	*P* value
**Male**			0.01
Rest	0.22	0.02	
Ringing	0.23	0.03	
Talking	0.24	0.02	
**Female**			0.1
Rest	0.22	0.03	
Ringing	0.21	0.03	
Talking	0.21	0.03	

**Table 2.** The Comparison of R Wave Voltage of Electrocardiogram in Different Studied Group (*P* value for Comparing Resting State with Talking Time)

	Mean, mv	Standard Deviation	*P* value
**Male**			0.7
Rest	0.5	0.2	
Ringing	0.51	0.2	
Talking	0.5	0.3	
**Female**			0.2
Rest	0.52	0.16	
Ringing	0.53	0.17	
Talking	0.52	0.18	

**Table 3.** The Comparison of QRS Complex Time in Different Studied Groups (*P* value for Comparing Resting State With Talking Time)

	Mean, s	Standard Deviation	*P* value
**Male**			0.2
Rest	0.08	0.01	
Ringing	0.07	0.01	
Talking	0.07	0.01	
**Female**			0.1
Rest	0.07	0.01	
Ringing	0.07	0.01	
Talking	0.06	0.01	

## 5. Discussion

This study conducted to survey the effect of mobile electromagnetic waves on 40 Zahedan medical university students who had referred to the laboratory of physiology. In this study, talking through mobile phone caused significant increase of heart rate in both genders in comparison with resting and ringing states of mobile, this increase was considered as a sinus arrhythmia (*Figure 1*). In addition to internal electricity guidance system, heart rate was influenced by the activity of autonomic nervous system. Thus this effect can be the result of mobile phone waves on nervous system which is consistent with the result of Ahamed ****et al****. study (10). On the other hand, regarding the electricity square of heart, being adjacent to another electricity field may cause intervention and lead to heart arrhythmia. Another study on the effects of mobile phone on heart rate of students indicated significant difference in HRV during talking in comparison with before talking state which was in accordance with the current study. However in another study no significant difference on heart rate and electrocardiogram parameters of rats was reported under the influence of the mobile phone use. The effects of mobile phone waves on blood pressure and heart rate according to the studied subjects were reported differently in human being (10). Kaviannezad ****et al****. (11) reported the onset of sinus arrhythmia as the result of mobile phone waves which is in accordance with the current study. It seems that different methods in these studies caused different results. Also the brand of mobile phone may influence on the results. In this study TP segment during talking had a significant difference in males in comparison to resting and ringing states, where as TP segment in females had significant difference in all three states (*Figure 2*). It seems that significant decrease of TP segment is the main cause of heart rate increase during talking through mobile phone, although the effect of motivation caused by ringing and talking should not be ignored. PR interval, Time of QRS wave and voltage of R wave didn’t have any significant differences in all three states. It is probable that the effects of mobile waves on PR interval and QRS complex time was not measurable due to short time of these parts. In male group, T wave time during talking in comparison to resting and ringing states indicated significant increase whereas in females it didn’t show any significant difference (*Table 1*). Length of T wave time can affect heart arithmetic, although in this study disturbances of heart rhythm was not considered. No significant difference was observed in electrocardiogram parameters of rats exposed to mobile phone (5).Probably this is because of the difference of heart rate in human beings and rats, the high heart rate of rats makes measuring the changes impossible. The results of this study indicated that there was significant increase in heart rate of both genders because of electromagnetic waves of mobile phone. In regard to the findings of this study, it should be considered that long term and constant use of mobile phone may cause harmful effects on heart. Additionally several reports regarding the effects of these waves on biological processes deserve caution when using mobiles, for example using them less often and putting the device far from body also using mobiles by children and teenagers should be avoided as much as possible.
